# Editorial

**Published:** 1890-02

**Authors:** 


### Editorial.

### A FEW PRACTICAL HINTS.

The goddess of health wields her sceptre over but few loyal and willing subjects. Her empire has but slowly increased since it began in the days of Moses. Since his time there has been a great neglect of the laws of sanitation, and millions of people have died therefrom. The germs of disease are scattered everywhere, and it is the duty of all to protect themselves, their own homes, their villages, towns and cities against their invasion. Personal hygiene is practiced by the better classes in most cases, but there is a woeful non-observance by those of the lower. The people need information on this subject. They should be taught that it is better to neglect any other ordinary duty of life. Indeed, the laws of personal hygiene should be religiously observed. This would not only bring health and happiness to us, but to posterity.

Those living in the country have good opportunities to enjoy healthy homes, if they but knew how to make and keep them so. There is astonishing ignorance, among them, on this subject. The farmer frequently has his hog-pen a short distance from his dwelling, and the foul odors are wafted into it by every passing breeze. The women wash the dirty clothes around the well, and suds laden with filth find their way to the bottom and poison the family, and typhoid fever, diphtheria, dysentery or some other malignant disorder will be the result. These two vile causes of disease and death are enough for us to mention at this time, and we believe that it is the duty of every physician to sound the alarm in the rounds of his practice, not only when these but when any other violation of sanitary science is known to him. In fact the public should be forewarned against the direful consequences which follow the transgression of plain and conservative laws, which, if strictly and intelligently carried out, would rob the grave of so many victims.

In the city of West Point, State of Georgia, in the summer of 1881, we had a large number of cases of typho-malarial fever, and were at a great loss to divine the cause. There are two sluggish branches which meander through the east side of the city, and there were quite a number of privies on these or over them. There was also a ditch over which there were half a dozen privies used by negro families. The branches and ditch were so dried up that the water stood in pools along the deeper places. One day when my horse was hot and thirsty I rode him into one of these pools, and he snorted and refused to taste the water. Just above, on the side of the hill near the pool, stood a privy. This was a key to the cause of our fever. I at once made a full investigation and found matters as before stated.

Allow me to say that the city is situated on either side of the Chattahoochee river, into which these branches, when running, empty. The city fathers were immediately notified and an ordinance was passed to abate the nuisances. Some of the owners kicked, and by inexcusable neglect the order was not executed.

But I took time by the forelock the next season and had some of the privies moved and others torn down, very much to the displeasure of some of my best personal friends. I am proud to say that we had but two or three sporadic cases during that season, and there were no deaths. During the summer of 1887 typho malarial fever broke out in Bluffton, a suburb of West Point. About fifty yards from a house in which there were three fearful cases of the fever, (white people), there were three or four large hogs in a close pen, fed mostly on slops. There were also two serious cases of the fever in the house just beyond, these were negroes. Now, every case that occurred, and there were a good many, could be directly traced to hog-pen poisoning. Two or three years ago I was called in consultation to see four bad cases of typhoid fever in Alabama. Upon investigation I found that washing was done around the well. There were several stumps around the same, and my conclusion was, that the dead roots helped to convey the washings into the well. This well was situated right at the horse-lot, and several fine hogs were in the lot, and had a wallow but a short distance below, on ground a little lower than that at the top of the well. I advised them to send elsewhere to get drinking water.

This subject is inexhaustible and I have brought it forward because of its great importance to science and humanity, and because the physician should be a watchman on the tower to give alarm when he sees the enemy approaching. A. W. G.

---

### DEATHS FROM ETHER.

---

Two deaths from the administration of ether as an anæsthetic have been reported within the last month; one in Bellevue Hospital, and the other in the city of Atlanta. In the presence of these and similar reports that startle the country from time to time, it well becomes us to ask the question, is this anæsthetic as free from danger as it is believed? It has been long held by those who advocate it that there is practically no danger in it, but the facts are very far from bearing it out, and it is a serious question as to whether, in the end, it is in the aggregate any less hazardous than its much dreaded brother chloroform. At least it may well be said that no operator is justified in assuring any patient that ether anæsthetic is absolutely free from danger.

In each of the cases reported it is presumed that the administration was in the hands of experienced etherizers, and that all was done that should be to guard against danger, so that the anesthetic was rightly chargeable with the disastrous result. The moral taught is that all anesthetics are dangerous, and the patient has the right to know it.

---

### DR. J. B. S. HOLMES.

---

The commuuity of Rome, and indeed the entire State, has been recently shocked by the fearful tragedy which resulted in the killing of Mr. DeForest Allgood by his brother-in-law, Dr. J. B. S. Holmes. We rejoice to see from the report of the investigation of the coroner's jury, as well as the verdict of the people of his home, that the doctor is freed from all blame in the matter, and that he was acting purely in self-defense. But in its best light the occurrence is awfully tragic for the unfortunate gentleman who was unwillingly forced to the deed.

No physician in the State stands higher than Dr. Holmes. The leader of the profession in his section, the President of the Medical Association of Georgia, and possessing a personality that always charms, he numbers his friends and admirers, in and out of the profession, by the thousand. In the severe trial that has been forced upon him, he has the sincere symyathy of his professional friends, especially, and THE RECORD extends to him its unqualified respect, sympathy and esteem.

---

Owing to the absence of Dr. W. F. Westmoreland, Jr., the second part of his paper could not come out in this number. It will appear in the March issue.

THE latest aspirant for recognition in medical journalism is the *Dixie Doctor*, edited by T. H. Huzza, M. D.

It is a neat, sprightly looking journal, printed on fine paper and in excellent style.

The editor has set a high standard, and we hope and believe it will be maintained. We shall watch with interest the success of this journal.

---

WE learn with regret of the death of Dr. Lewis H. Sayre, son of the celebrated surgeon, Lewis A. Sayre.

He was a young man of great promise, and already enjoyed a lucrative practice. He graduated at Bellevue in 1876, and was thirty-eight years old. His death was caused by heart failure.

